# Novel De Novo *RALA* Missense Variants Expand the Genotype Spectrum of Hiatt‐Neu‐Cooper Neurodevelopmental Syndrome

**DOI:** 10.1002/mgg3.70072

**Published:** 2025-02-07

**Authors:** Alice Dainelli, Mohammad Sadegh Shams Nosrati, Ferruccio Romano, Fabiana Vercellino, Maria Margherita Mancardi, Annalaura Torella, Vincenzo Nigro, Valeria Capra, Federico Zara, Marcello Scala

**Affiliations:** ^1^ Department of Neuroscience, Rehabilitation, Ophthalmology, Genetics, Maternal and Child Health (DINOGMI) University of Genoa Genoa Italy; ^2^ Medical Genetics Unit IRCCS Istituto Giannina Gaslini Genoa Italy; ^3^ Genomics and Clinical Genetics IRCCS Istituto Giannina Gaslini Genoa Italy; ^4^ Child Neuropsychiatry Unit SS Antonio e Biagio e Cesare Arrigo Hospital Alessandria Italy; ^5^ Unit of Child Neuropsychiatry, IRCCS Istituto Giannina Gaslini Epicare Network for Rare Disease Genoa Italy; ^6^ Department of Precision Medicine University of Campania “Luigi Vanvitelli” Naples Italy; ^7^ Telethon Institute of Genetics and Medicine Pozzuoli Italy

**Keywords:** brain abnormalities, epilepsy, exome sequencing, GTPase, intellectual disability, neurodevelopmental syndrome, RALA

## Abstract

**Background:**

RALA is a small GTPase from the RAS superfamily implicated in signal transduction and cytoskeletal dynamics. Recently, de novo variants in *RALA* have been associated with a neurodevelopmental syndrome characterized by intellectual disability (ID), developmental delay (DD), and seizures. So far, only < 12 patients have been reported.

**Methods:**

In this study, we report two novel patients with neurodevelopmental impairment and epilepsy carrying previously unreported *RALA* variants. We performed a thorough clinical investigation of these patients and performed brain MRI to detect potential abnormalities. Trio‐exome sequencing and/or NGS panel testing were conducted to identify the genetic variants. Then, we reviewed previous cases reported in the literature.

**Results:**

Affected individuals showed a complex neurodevelopmental phenotype consistent with Hiatt‐Neu‐Cooper neurodevelopmental syndrome. Brain MRI in both subjects showed abnormalities including megalencephaly and ventricular enlargement, previously unreported in *RALA* patients. Genetic testing revealed two novel de novo missense variants in *RALA*: c.217G>A, p.(Glu73Lys) in case #1 and c.73G>C, p.(Val25Leu) in case #2. Both variants affect highly conserved residues within the GTP/GDP‐binding site of the protein. These changes are predicted to be deleterious by in silico tools, interfering with the GTPase activity of RALA.

**Conclusion:**

Our findings expand the genotype and phenotype spectrum of Hiatt‐Neu‐Cooper neurodevelopmental syndrome. Our observations also support the important role of variants affecting the GTP/GDP‐binding site of the RALA protein in the pathogenesis of Hiatt‐Neu‐Cooper neurodevelopmental syndrome.

## Graphical abstract

RALA is a small GTPase from the RAS superfamily involved in signal transduction and cytoskeletal dynamics. In this study, we identified two novel de novo missense variants in RALA (c.217G>A p.(Glu73Lys) and c.73G>C p.(Val25Leu)) in patients with developmental delay, epilepsy, and brain abnormalities. These rare variants affect conserved residues within the GTP/GDP‐binding site, likely disrupting RALA's GTPase activity, which is crucial for neurodevelopment.

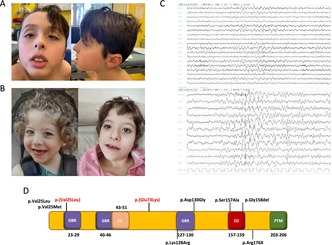

## 1 Introduction

*RALA* is an essential gene that belongs to the RAS superfamily of small GTPases, essential mediators of a variety of cellular activities, including signal transduction pathways and cytoskeletal dynamics (Sugihara et al. 2002; Scala et al. 2021; Tzima 2006). The fine regulation of these delicate processes is crucial for the development of the brain, allowing the proper migration, maturation, and function of neuronal cells (Sugihara et al. 2002; Nishiyama 2019; Scala et al. 2022). The *RALA* gene encodes Ras‐related protein Ral‐A, which functions as a molecular switch cycling between an inactive GDP‐bound state and an active GTP‐bound state (Sugihara et al. 2002; Scala et al. 2021). Activation of RALA occurs through GDP–GTP exchange, facilitated by guanine nucleotide exchange factors (GEFs), and is terminated by GTP hydrolysis, catalyzed by GTPase‐activating proteins (GAPs) (Sugihara et al. 2002; Scala et al. 2021). GEFs and GAPs are crucial actors in the GDP–GTP exchange cycle and their dysfunction has been implicated in several disorders, including neurodevelopmental syndromes featuring epilepsy and cognitive impairment (Scala et al. 2021, 2022, 2024; Katsanevaki et al. 2024).

RALA is primarily involved in intracellular signaling pathways that regulate crucial cellular processes such as proliferation, survival, and migration (Sugihara et al. 2002; Tzima 2006). It interacts with various effector proteins, including the exocyst complex, phospholipase D1, and the serine/threonine kinase STK33. Through these interactions, RALA regulates vesicle trafficking, actin cytoskeleton remodeling, and cell adhesion dynamics (Sugihara et al. 2002; Tzima 2006). The dysregulation of RALA has been implicated in several diseases, particularly cancer (Lim et al. 2005; Wagner et al. 2020; Martin et al. 2014; Ginn et al. 2016; Richardson et al. 2022; Wu et al. 2013).

In humans, deleterious variants in *RALA* cause a complex syndrome characterized by variable phenotypical features including global developmental delay, intellectual disability, delay or inability to walk, absent or delayed speech, autism spectrum disorder (ASD), seizures, exotropia, and dysmorphisms (MIM # 619311). In this study, we described two novel patients harboring variants in *RALA*, expanding the genotype–phenotype spectrum of this emerging condition (Wagner et al. 2020).

## 2 Case Presentation

### 2.1 Case #1

**FIGURE 1:**
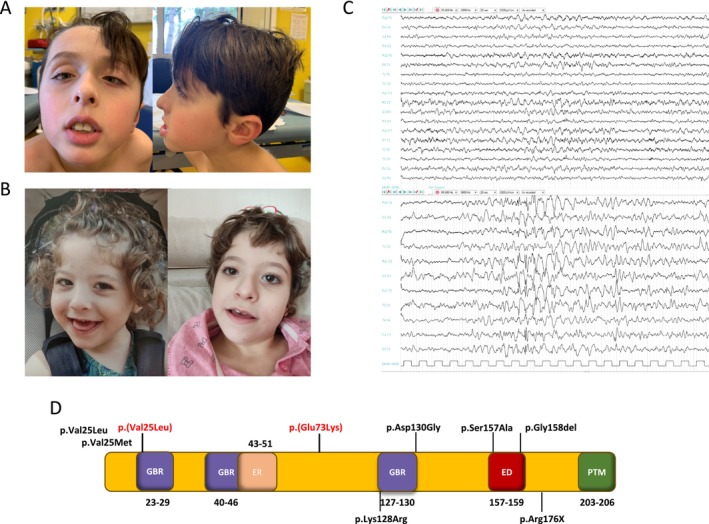
Clinical and genetic findings. (A) Frontal and lateral view of the facial features of patient 1, showing broad forehead, triangular face, down‐slanting palpebral fissures, exotropia, mandibular hypoplasia with pointed chin, and low‐set ears. (B) Patient 2 showing dysmorphic features: Mild epicanthus, short nose with flattened nasal bridge, slightly anteverted nares, and mandibular hypoplasia with pointed chin. (C) EEG showing disorganized posterior rhythm with superimposed slow anterior activity and high‐voltage bilateral spikes, polispikes, and sharp waves, predominantly over the temporal and central regions, enhanced during sleep. (D) Linear structure of the RALA protein, showing the GTP/GDP‐binding regions (GBR), effector region (ER), effector domain (ED), and post‐translational modification regions (PTM). The GBR is essential for the protein's molecular switch activity, while the ER and ED mediate interactions with downstream effectors. PTMs regulate protein activity, localization, and interactions. Variants identified in our patients and in prior studies are highlighted to illustrate their positions within these functionally critical regions.

A 10‐year‐old boy, born to unrelated healthy parents of Italian ancestry. Family history was negative for neurodevelopmental and neurological disorders. He was born at 39 gestational weeks, with normal birth parameters and regular neonatal course. Developmental milestones were achieved with mild delay. He was able to walk without support by the age of 19 months. An ophthalmological examination revealed alternating exotropia, treated with occlusions (Figure 1A). At the age of 7, the patient developed tonic–clonic generalized seizures. Interictal EEG revealed bilateral parieto‐occipital epileptiform abnormalities. Antiseizure treatment with valproic acid was started with an initial good response. However, seizures relapsed 1 year later, with a focal onset and secondary generalization into bilateral motor seizures. These episodes presented on a weekly basis and were only partially controlled after increasing the dosage of valproic acid. Brain MRI showed megalencephaly associated with a subcortical white matter hyperintensity in the right cerebellar hemisphere, minor hyperintensities in peritrigonal regions bilaterally, and an intrasellar arachonoidocele suggestive of a partial empty sella. Follow‐up neuropsychiatric evaluations led to a diagnosis of autism spectrum disorder with expressive communication skills limited to non‐verbal vocalizations, but preserved comprehension of simple sentences. Some episodes of aggressive behavior were reported. The patient was able to attend middle school with support and weekly sessions of applied behavioral analysis therapy. Physical examination at the age of 10 years revealed dysmorphic features with macrocephaly (55.6 cm, 2.48 SD), broad forehead, triangular face, down‐slanting palpebral fissures, mandibular hypoplasia with pointed chin, and low‐set ears. He also showed a hyperkinetic features, verbal and motor stereotypies, and flat feet. Karyotype yielded to negative results, whereas array comparative genomic hybridization analysis (aCGH) led to identification of a benign paternally inherited duplication in 10q26.3.

### 2.2 Case #2

A second child of healthy unrelated parents of Italian ancestry. Family history was negative for neurological or psychiatric diseases. Pregnancy was complicated by polyhydramnios. The patient was born at term by caesarean section, with normal birth parameters. At birth, muscular hypotonia was observed on physical examination. At the age of 7 months, the patient was diagnosed with developmental delay. She had an inadequate head control and was unable to sit due to severe hypotonia. She had social smile and could make vocalizations. Physical examination showed dysmorphic facial features, including mild epicanthus, a short nose with a flattened nasal bridge and slightly anteverted nares, mandibular hypoplasia with pointed chin (Figure 1B). Electroencephalograms (EEGs) showed a disorganized posterior rhythm with superimposed slow anterior activity and high voltage bilateral spikes, polyspikes and sharp waves especially over the temporal and central regions enhanced by sleep (Figure 1C). Brain MRI revealed enlargement of pericerebral frontal and temporal spaces (Figure S1). At the age of 9 years, presented with a prolonged tonic–clonic seizure, with eyes and head deviation to the right. Interictal EEG revealed bilateral fronto‐centro‐temporal epileptiform abnormalities, more prominent in the left hemisphere. Antiseizure treatment with levetiracetam was started with seizure control. At the last examination, at the age of 10 years, she had severe axial hypotonia and scoliosis, head but not trunk control, a profound intellectual disability, limited use of hands and absent speech. Feeding difficulties and some episodes of self and hetero‐aggressive behavior were reported. Karyotype and aCGH yielded negative results. Methylation test for uniparental disomy was also negative.

### 2.3 Genetic Analyses

For genetic testing, genomic DNA was extracted from peripheral blood leukocytes for exome sequencing (ES) (case #1) or next‐generation sequencing (NGS) panel testing (case #2) using standard local protocols (Katsanevaki et al. 2024). The identified variants were filtered out according to a minor allele frequency (MAF) of ≤ 0.001 in the gnomAD dataset (v4.1.0). Candidate variants were selected based on the conservation of the affected residues (GERP score) and the in silico predictions according to the ensemble variant effect predictor (VEP) pipeline, which incorporates combined annotation dependent depletion (CADD), sorting intolerant from tolerant (SIFT), and polyphen‐2 algorithms (Scala et al. 2018). The variants were then classified according to the American College of Medical Genetics and Genomics and the Association for Molecular Pathology (ACMG/AMP) guidelines. *RALA* variants were reported based on the RefSeq transcript NM_005402.4, following HGVS recommendations. In case #1, ES performed in the family trio led to the identification of the de novo c.217G>A p.(Glu73Lys) variant in the *RALA* gene. This variant is absent in gnomAD and affects a highly conserved residue in close proximity with a GTP/GDP binding site (amino acidic residues 70–71, Figure 1). It is classified as a VUS according to ACMG/AMP guidelines, though computational prediction tools unanimously support a deleterious effect on the gene (Table S1). In case #2, two different NGS panels were performed. The first was a 221 gene NGS panel including genes involved in a broad spectrum of neurodevelopmental disorders, that led to no pathological results. A second panel was later performed, extended to 705 neurodevelopmental genes. This test revealed the de novo missense variant c.73G>C, p.(Val25Leu) in *RALA*. The variant is absent in gnomAD and affects a conserved residue within the GTP/GDP‐binding site domain (amino acidic residues 23–29). It is predicted deleterious by in silico tools (Table S1) and it is classified as pathogenic according to ACMG/AMP guidelines.

## 3 Discussion

Deleterious variants in the *RALA* gene have recently been associated with a syndrome characterized by neurodevelopmental and musculoskeletal abnormalities (Table 1) (Sugihara et al. 2002; Scala et al. 2021; Hiatt et al. 2018; Okamoto et al. 2019) This rare genetic disorder caused by *RALA* gene variants affects various aspects of an individual's development such as developmental delay, intellectual disability, and speech impairment (Sugihara et al. 2002; Scala et al. 2021; Tzima 2006; Nishiyama 2019). They may also exhibit distinctive facial features, including a prominent forehead, deep‐set eyes, and a broad nasal bridge (Sugihara et al. 2002; Scala et al. 2021). Furthermore, musculoskeletal anomalies such as joint laxity, scoliosis, and contractures have been observed (Sugihara et al. 2002; Scala et al. 2021). Prior studies have revealed that *RALA* gene variants lead to disruption in critical cellular processes and signaling pathways involved in neurodevelopment (Nishiyama 2019; Wagner et al. 2020; Nussinov et al. 2022). RALA plays a vital role in regulating intracellular signaling and cytoskeletal dynamics, which are essential for neuronal development, synaptogenesis, and synaptic plasticity (Nishiyama 2019; Wagner et al. 2020). Disruptions in these processes resulting from *RALA* variants can lead to abnormal brain development and impaired cognitive function.

**TABLE 1:** Summary of clinical features of *RALA* patients.

Patient	RALA variant (NM_005402.4)	Amino acidic change	Gender	Abnormal OFC	Developmental delay	Cognitive disability	Speech	ASD	Hypotonia	Ability to walk	Seizures	Dysmorphism	Brain MRI anomalies
Patient 1	c.73G>A	p.(Val25Met)	F	NR	+	Moderate	Delayed	+	−	+	+	+	−
Patient 2	c.73G>A	p.(Val25Met)	M	−	+	Severe	Absent	+	+	−	−	+	+
Patient 3	c.73G>A	p.(Val25Met)	M	−	+	Severe	Delayed	+	+	+	−	−	−
Patient 4*	c.73G>T	p.(Val25Leu)	M	−	+	Profound	Absent	NR	+	−	+	+	+
Patient 5*	c.73G>T	p.(Val25Leu)	M	−	+	Profound	Absent	NR	+	−	+	+	+
Patient 6	c.383A>G	p.(Lys128Arg)	M	−	+	Severe	Absent	NR	+	−	+	+	+
Patient 7	c.383A>G	p.(Lys128Arg)	F	−	+	Severe	Absent	NR	+	−	−	+	+
Patient 8	c.389A>G	p.(Asp130Gly)	M	−	+	Severe	Absent	NR	+	−	+	−	+
Patient 9	c.469T>G	p.(Ser157Ala)	M	−	+	Severe	Delayed	NR	+	+	−	+	+
Patient 10	c.472_474delGCT	p.(Gly158del)	M	−	+	Severe	Delayed	NR	+	−	−	+	+
Patient 11	c.526C>T	p.(Arg176*)	M	Macrocephaly	+	Profound	Absent	NR	+	−	+	+	+
Patient 12	c.73G>A	p.(Val25Met)	F	Microcephaly	+	Severe	Absent	NR	+	+	NR	+	−
Subject 1 (our study)	c.217G>A	p.(Glu73Lys)	M	Macrocephaly	+	Severe	Absent	+	−	+	+	+	+
Subject 2 (our study)	c.73G>C	p.(Val25Leu)	F	−	+	Severe	Absent	−	+	−	−	+	+

*Note:* Patients 1–11 are taken from Hiatt et al. (2018), patient 12 from Okamoto et al. (2019).

Abbreviations: ASD, autism spectrum disorder; HC, head circumference; MRI, magnetic resonance imaging; OFC, occipitofrontal circumference; pc, percentile.

Developmental delay and intellectual disability have been consistently observed in all reported RALA patients, with variability in individual developmental trajectories. Language delays are common, with 53% of patients exhibiting absent speech or severe language delays. ASD was diagnosed in one‐third of patients. In these cases, speech impairment was severe and likely cumulative, resulting from developmental delay and autistic phenotypes. Regarding motor development, 80% of patients presented with hypotonia at birth or during early development, with marked variability in individual motor outcomes. While five patients achieved autonomous, though unsteady, walking between 2 and 3 years of age, 64% never attained this milestone. In these patients, there were persistent central hypotonia and mild peripheral spasticity. Feeding difficulties were noted in a subset of patients, with some requiring gastrostomy. (Hiatt et al. 2018) Epilepsy was reported in 60% of patients, featuring either focal and generalized seizures, with age at onset ranging from infancy to late childhood. Notably, no correlation was observed between epilepsy and other clinical features, such as motor development or ASD. Neuroradiologic findings were variable in previous cases, including corpus callosum hypoplasia and delayed myelination, while some patients had normal brain MRI. Overall, these findings emphasize the heterogeneous clinical presentation in RALA patients, with some shared features but no pathognomonic markers (Hiatt et al. 2018).

Certain clinical features appear to be more consistent among the cases described so far, including our case 2: upslanting palpebral fissures, mild epicanthus, a short nose with a flattened nasal bridge, slightly anteverted nares, prominent forehead, horizontal eyebrows, mild ptosis, wide nasal bridge, short philtrum, thin upper lip vermillion with an exaggerated Cupid's bow, pointed chin, and low‐set ears with increased posterior angulation (Hiatt et al. 2018). Macrocephaly is also commonly reported (Hiatt et al. 2018; Okamoto et al. 2019). Our cases share several features with previously described patients with *RALA* pathogenic variants (Hiatt et al. 2018), though also presenting some less typical findings as well. The clinical presentation of case 2, characterized by hypotonia, feeding difficulties, developmental delay, and distinct facial dysmorphism aligns with the core phenotype for *RALA* variants. Similarly, the clinical features of subject 1, including intellectual disability, language delay, behavioral issues, exotropia, seizures, and characteristic dysmorphic traits, are consistent with the border spectrum of RALA‐related conditions. Additionally, previously unreported neuroradiological findings such as partial empty sella in case 1 and ventricular enlargement in case 2 may contribute to expand the phenotypic spectrum associated with *RALA* variants. Further cases with detailed neuroradiological descriptions are needed to better understand brain malformations in RALA patients.

In this study, we report two new patients with the Hiatt‐Neu‐Cooper neurodevelopmental syndrome harboring novel de novo variants in the *RALA* gene. The missense variants broaden the genotype spectrum of this complex syndrome, although no clear genotype–phenotype correlations were identified in our patients. Regarding the disease mechanisms, it is thought that developmental delay is not caused by a simple loss‐of‐function of RALA. First, no clearly pathogenic loss‐of‐function (such as nonsense, frameshift, splice‐site) have been reported to GeneMatcher or in the literature to date (Hiatt et al. 2018). Second, studies in mice show that heterozygosity for loss of function does not significantly affect development or viability. Third, the de novo missense variants described by Hiatt are enriched in residues involved in GTP/GDP‐binding, with six alleles recurring at only two codons. Fourth, missense *RALA* variants in the general population are located outside of these critical GTP/GDP‐binding regions, indicating selective depletion similar to that observed in other small GTP‐binding proteins (Scala et al. 2024; Katsanevaki et al. 2024; Tran et al. 2022). Finally, multiple disease‐associated RALA positions observed by Hiatt are homologous to positions at which variants in other small GTPases known to disrupt GTPase activity and cause disease. Collectively, these findings suggest that reduced RALA dosage alone is not inherently pathogenic, rather, disease likely arises from specific alteration in GTP/GDP‐binding dynamics (Katsanevaki et al. 2024; Tran et al. 2022). Functional assays have confirmed that all identified variants result in reduced GTPase activity (Hiatt et al. 2018).

The c.73G>C, p.(Val25Leu) variant identified in case 2 affects a recurrently implicated amino acidic residue. In Hiatt's cohort, 5 of 11 probands harbored variants involving the 25th residue while Val25 does not directly interact with GTP/GDP, variations at this position (p.(Val25Met) and p.(Val25Leu)) are likely to disrupt the structure of the GTP/GDP‐binding pocket, as suggested by functional assays (Hiatt et al. 2018). Additionally, the c.217G>A, p.(Glu73Lys) variant in case 1 is located near a GTP/GDP‐binding site (70–71 residues), likely affecting the function of the adjacent binding site. Functional assays from Hiatt et al. revealed reduced GTPase activity across all tested variants but variability in effector binding (Hiatt et al. 2018). One variant exhibited increased effector binding, a characteristic of oncogenic RAS alleles, while others showed reduced effector binding. This suggests a divergent functional impact rather than a uniform mechanism, such as a dominant‐negative effect. Furthermore, genotype–phenotype correlations were inconsistent (Hiatt et al. 2018). Individuals with identical variants (e.g., p.(Val25Met) or p.(Val25Leu)) displayed differing phenotypes, such as presence or absence of seizures. These findings highlight the complexity of the pathophysiology of RALA‐related disorder and suggest that reduced GTPase activity and altered GTP/GDP‐binding dynamics underlie disease mechanisms without necessarily implying a dominant‐negative effect. Additional functional and clinical studies are needed to further clarify these mechanisms and explore the potential role of genetic, environmental, and stochastic factors in phenotype variability.

In summary, we reported two novel patients with Hiatt‐Neu‐Cooper neurodevelopmental syndrome harboring two previously unreported missense variants in the *RALA* gene. Our study expands the genotype and phenotype spectrum of this very rare condition, suggesting that variants affecting the GTP/GDP‐binding site of the RALA protein may be especially relevant for the pathophysiology of this very rare condition. Further investigations aiming at unravelling the functional impact of disease‐causing *RALA* variants will offer important insights into the molecular pathways underlying human neurodevelopmental disorders.

## Author Contributions

The author takes full responsibility for this article.

## Ethics Statement

This study adheres to the principles in the Declaration of Helsinki. The study was reviewed by IRCCS Istituto Giannina Gaslini Review Board (IRB) (Comitato Etico della Regione Liguria, protocol 163/2018) protocol. Written informed consent was obtained from all participants including consent for publication of photographs as required by the IRB. Consent forms are archived and available upon request.

## Supporting information


Data S1:

Data S2:

## Data Availability

The data that support the findings of this study are available from the corresponding author upon reasonable request.
