# Supplementary material for: Novel De Novo RALA Missense Variants Expand the Genotype Spectrum of Hiatt‐Neu‐Cooper Neurodevelopmental Syndrome
Source: Mol Genet Genomic Med. 2025 Feb 7;13(2):e70072. doi: 10.1002/mgg3.70072 (PMC11803908; doi:10.1002/mgg3.70072)
Supplement: Supplementary file 1 — Data S1: [file MGG3-13-e70072-s002.docx]

**Supplemental Material**

**1. Supplemental Figures**

**2. Supplemental Tables**

**1. Supplemental Figures**

**
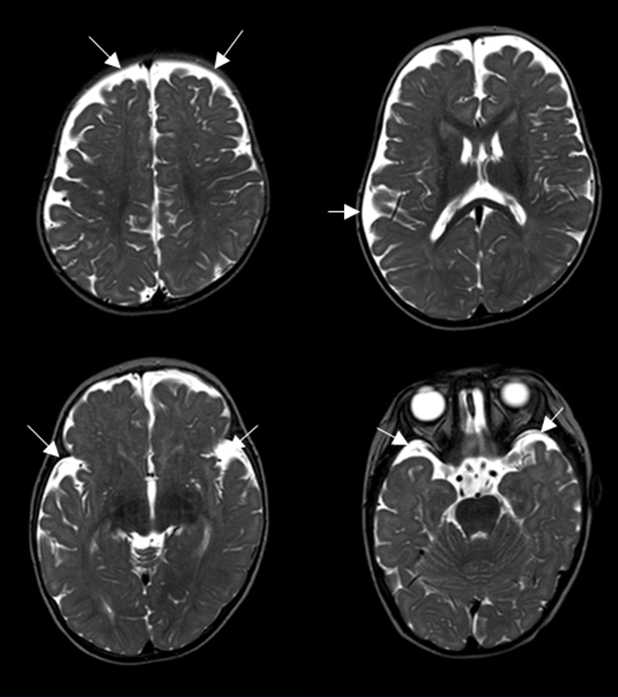
**

**Figure S1. Brain MRI of patient 2.** T2-weighted axial scans showing enlargement of the pericerebral frontal, parietal, and temporal subarachnoid spaces, bilaterally.

**2. Supplemental Tables**

**Table S1.** *In silico* analysis of the *RALA* variants identified in our cases.
